# Downstream Changes in Odonate (Insecta: Odonata) Communities along a Suburban to Urban Gradient: Untangling Natural and Anthropogenic Effects

**DOI:** 10.3390/insects12030201

**Published:** 2021-02-27

**Authors:** Wade B. Worthen, R. Kile Fravel, Connor P. Horne

**Affiliations:** 1 Biology Department, Furman University, Greenville, SC 29613, USA; 2 Independent Researcher, 1716 Johnson Marina Rd, Chapin, SC 29036, USA; kile.fravel@gmail.com; 3 School of Medicine, University of South Carolina, Greenville, SC 29605, USA; cphorne@email.sc.edu

**Keywords:** Odonata, dragonfly, anthropogenic effects, community ecology, nestedness

## Abstract

**Simple Summary:**

Dragonflies are sensitive to natural and human-caused variation in the aquatic and terrestrial habitats where their larvae and adults live. For example, a reduction in shady vegetation, as a consequence of increasing stream size or streamside deforestation, often causes a reduction in specialized forest species and an increase in generalist species. We surveyed larvae and adults at 15 sites along the Reedy River in Greenville Co, SC, USA, from headwater sites in forested suburban landscapes through the urban core of the city of Greenville. We described the sediment characteristics and shoreline vegetation in two 4 m × 20 m plots at each site, and measured the percentage of developed land, forested land, grasslands, and wetlands within 500 m of each plot center. At a small scale, within plots, larval abundance and diversity increased with increasing amounts of dead debris that may provide a refuge from predators. Adult abundance and diversity correlated with the amount of aquatic and shoreline vegetation used as perches. At a large scale, diversity responded more to natural changes in habitat than urbanization: damselfly diversity increased downstream and dragonfly diversity was greatest in sunny, open habitats with fields, wetlands, and open water.

**Abstract:**

The community structure of lotic odonates (Insecta: Odonata) changes downstream, but it is difficult to untangle natural and anthropogenic causes. We surveyed larvae and adults at 15 sites along the Reedy River in Greenville Co., SC, USA, from sites in forested suburban landscapes through the urban core of the city of Greenville. We used principal component analyses and Akaike information criteria models to describe the relationships between larval and adult community descriptors (abundance, richness, and diversity) and habitat characteristics at several spatial scales, including water chemistry, sediment and detritus, aquatic and streamside vegetation, and the percent cover of landforms in the surrounding landscape. At all scales, larval abundance, richness, and diversity correlated with the amount of detritus. At a small scale, adult indices correlated with the amount of sunlight and streamside vegetation. Zygopteran community composition was nested at a large scale; richness and diversity did not correlate with changes in the landscape but increased downstream. Anisopteran composition was also nested, but richness correlated with the percent cover of field, wetland, and open water in the habitat and was unrelated to downstream site position. Landscape transformation affected anisopterans more than zygopterans by opening habitats that facilitate these generalist heliotherms.

## 1. Introduction

Dragonflies (Insecta: Odonata) are excellent indicators of the ecological health of aquatic habitats and the surrounding landscape [1,2,3,4,5,6,7]. The species richness and diversity of both larval and adult communities, and the abundance of particular species that are sensitive to pollution or human disturbance, are useful indicators of anthropogenic impacts on freshwater systems [8,9,10,11,12]. In general, the most common negative impacts arise from pollution and declining water quality [9,13], the loss of aquatic and shoreline vegetation [14,15,16], reduction in the riparian zone [17,18,19,20,21,22,23,24], and loss of natural habitat in the surrounding landscape matrix [18,20,25]. Humans can have positive effects on odonate communities, though, by increasing the amount of habitat through ponds, canals, and wetland restoration [11,26,27,28], and increasing the quality of habitat by restoring aquatic and shoreline vegetation, meanders, and riparian zones [29,30]. Even large-scale structural changes that alter river flow, such as the presence of groynes and dredging [31,32], can have positive effects on odonate communities by increasing habitat heterogeneity and mediating other types of anthropogenic disturbance. 

Of course, preserving and restoring odonate communities require an understanding of the natural ecological drivers of diversity and the effects of particular anthropogenic impacts. Untangling these natural and human-induced patterns can be particularly difficult in streams and rivers, however, because both sets of factors may change downstream [33,34]. Nonetheless, considerable progress has been made on this front, with many studies from different climates and biomes showing similar patterns. Odonates in the suborder Zygoptera—perhaps because of their lower powers of dispersal and greater dependency on immediate environmental conditions [22,35]—tend to be negatively affected by anthropogenic change on a local scale [36,37,38]. They are more abundant and diverse in smaller streams with low pollution, a rich detritus base [20], and with a complete canopy in an intact forested matrix [22,39,40]. Odonates in the suborder Anisoptera tend to be affected by changes in the larger landscape [37,38]. They are more abundant in larger streams and rivers with more sunlight—either as a consequence of a naturally open canopy or habitat alteration and disturbance [16,19,39,40,41,42,43,44,45,46]. Thermal niche requirements may explain this difference; anisopterans are often heliotherms that need to bask to achieve suitable temperatures for flight, whereas zygopterans are often thermoconformers that can tolerate shaded areas and are less likely to overheat while basking [47]. These differences can lead to nested subset structure; shady habitats are dominated by small thermoconformers, while open sites also contain larger species limited to sunnier habitats [47].

The abundance and diversity of odonates also increases with the abundance of aquatic macrophytes [28,48,49,50] and intact shoreline vegetation. Adults perch on these structures for basking and for surveying their territories for mates, intruders, predators, and prey [51,52,53,54,55,56]. Some species also use aquatic macrophytes, algae, and detritus for oviposition [57], and larvae use these structures to hide from predators and search for food [58]. 

In this study, we describe the larval and adult odonate communities along a section of the Reedy River in Greenville County, SC, USA, examining which environmental variables—natural or anthropogenic—best explain changes in abundance, species richness, diversity, and nestedness at different spatial scales. This is an excellent area to study the impact of human land transformation, as the region has experienced the greatest net loss in forest cover in the Eastern USA in the last 40 years [59]. In a previous study conducted in the region, Worthen and Chamlee [60] found that adults were more abundant and diverse at lake and pond habitats than in streams and rivers, and communities in small habitats were nested subsets of those in large habitats. However, the effects of human development on odonate communities was difficult to assess across lentic and lotic habitats. Here, by sampling sites in one river—from forested headwaters in suburban areas through sites in an urban center—we parse the effects of anthropogenic landscape transformations and natural downstream changes on the abundance, richness, diversity, and nestedness of odonate assemblages. 

## 2. Materials and Methods

### 2.1. Sampling Protocol and Independent Variables

**Figure 1:**
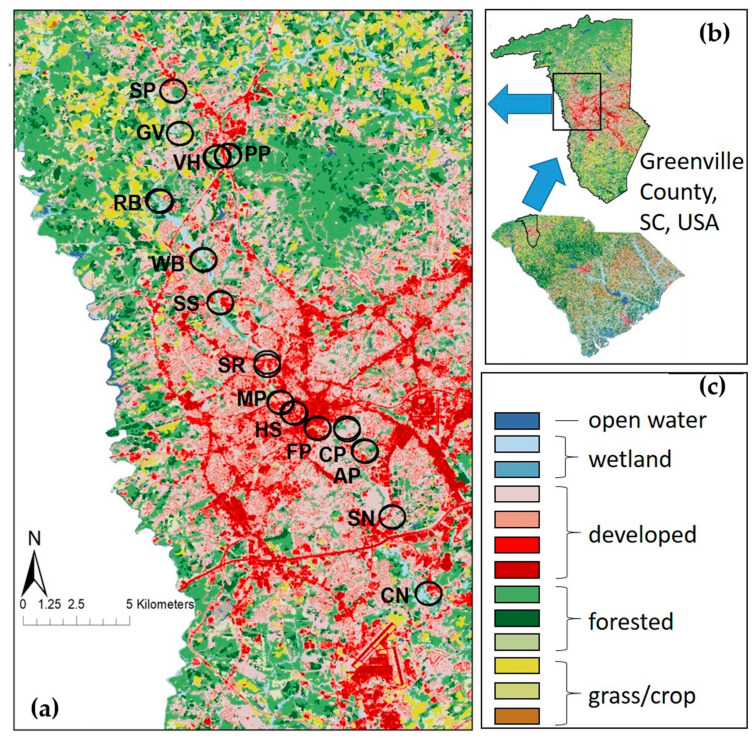
The location and landscape characteristics of study sites along the Reedy River in Greenville Co, SC, USA, showing: (**a**) landscape characteristics in circular samples (500 m radius) centered on each of two plots/site (upstream to downstream: Spring Park (SP), Green Valley Country Club (GV), Poinsett Park (PP), Vista House (VH), Riverbend Equestrian Park (RB), Watkins Bridge (WB), Sulphur Springs (SS), Swamp Rabbit Bakery (SR), Mayberry Park (MP), Hudson Street (HS), Falls Park (FP), Cleveland Park (CP), Andover Park (AP), South Pleasantburg Nursery (SN), and Lake Conestee Nature Preserve (CN)); (**b**) location of Reedy River in Greenville Co, SC, USA; (**c**) legend of color-coded land forms from the 2011 National Land Cover Database, grouped into categories used in this study.

From May to August 2019, we sampled adult and larval odonates at 15 sites along the Reedy River in Greenville Co, SC, USA (Figure 1), from two headwater streams in a suburban landscape through the urban core of the city of Greenville, SC. At each site, we established two 20 m × 4 m plots at least 10 m apart, each comprised of 10 2 m × 2 m terrestrial subplots along the bank adjoining 10 2 m × 2 m aquatic subplots in the river [60]. 

Larval odonates were sampled once at each site. We used a 1 m kick seine (3 mm mesh) and disturbed the substrate in a 1 m × 1 m plot on the upstream side of the net within each aquatic subplot for approximately 2 min. Although a kick seine is most appropriate for shallow riffle areas typical of headwater streams, we used it at all sites because we were sampling stream and river edges where the water was shallow (rarely > 30 cm deep) and the sediment a mixture of sand, rocks, cobble, and detritus. Larvae collected in the net were euthanized with hot water, transferred to 70% EtOH, and identified to genus in the laboratory. Adults were sampled three times at each site, approximately once a month to account for differences in flight seasons. We sampled on sunny days, from 10:00 to 14:00 [46], using a method similar to the “Odonata Scanning Protocol (OSP)” [61]. Two observers scanned each subplot and recorded the number of individuals of each species. In rare instances, individuals that could not be identified with binoculars (Pentax Papilio^©^ 8.5 × 21) were captured by net and identified in hand. These small subplots could be surveyed rapidly (within 1–2 min), reducing the incidence of immigration/emigration [59]. Nevertheless, we noted any individuals that moved to the next subplot and did not double count them. As in the OSP, data from subplots can be aggregated at the plot and sites scales to examine patterns at these scales [61]. In addition to these ‘scan’ surveys, we recorded additional species seen within the plot (but not in the subplot being sampled at the moment) and at the site (but not within a sampling plot) for species richness analyses at the plot and site scales, respectively. Data from the three samples were pooled before analysis.

Habitat characteristics were described at three spatial scales: subplot (2 m), plot (20 m), and site (1 km). At the subplot scale, the percent cover of different vegetation forms (none, less than 20 cm, 20–100 cm, 100–200 cm, 200–300 cm) was calculated by drawing the regions of different sized vegetation on subplot maps and then calculating the percent cover of these areas from overhead photographs using ImageJ^©^ software [62]. Likewise, the percent cover of the following substrates in aquatic subplots was calculated by averaging estimates of two observers: bedrock, cobble, gravel, sand, mud, algae, rooted macrophytes, and detritus (recognizably organic benthic debris such as leaf litter, sticks, and logs). These estimates were made once for each subplot. We also estimated the percent cover of sunflecks in each terrestrial subplot on three sampling dates, at different times of day to account for differences in diurnal angle, and used the mean value for analysis. We described the habitat at the plot scale three ways. First, we averaged the subplot percent cover values for each plot. Second, we used a YSI model 85^©^ meter to measure water temperature (°C), pH, conductivity (µS/cm), and dissolved oxygen (mg/L) at subplots 1, 5, and 10 in each plot, and averaged these values to characterize these parameters per plot. These measurements were taken once, when larvae were sampled. Third, the percentage of different landforms within 500 m of the center of each plot were calculated using ArcGIS [63] and the 2011 National Land Cover Database (NLCD) [64,65]. Landform categories were combined into five categories (open water, development, forest, grassland, and wetland) for statistical analysis. At the site scale, habitat characteristics were computed by averaging the metrics in the plots sampled at each site. Recognizing that a host of other physicochemical variables change downstream, we also included the variable “downstream position” which ranked the sites downstream from the two headwaters (rank = 1) to the farthest site downstream (rank = 13). All percent cover values were arcsin square root transformed for analysis.

### 2.2. Dependent Variables

We calculated three community indices for larvae at the subplot, plot, and site scales: total abundance of larvae, genus richness, and genus diversity (calculated by Simpson’s reciprocal diversity = 1/Ʃ(p_i_^2^), where p_i_ = proportion of individuals in a sample in the i^th^ genus). For adults, since zygopterans and anisopterans respond differently to habitat characteristics, we calculated abundance, species richness, and species diversity at the subplot, plot, and site scales for zygopterans, anisopterans, and all odonates. Although the abundance values at larger scales are just the sums of values at lower scales, richness and diversity values are dependent upon the distribution of abundances across particular species and so are neither sums nor averages of values at smaller scales. In addition, richness values at the plot and site scales include those additional sightings mentioned above.

### 2.3. Statistical Analyses 

All statistical analyses were performed using SPSS v. 23 [66]. We used nested linear models to determine whether mean abundance (log_10_ transformed), richness, and diversity of larvae and adults in subplots varied among plots and sites. We used Akaike information criteria models (AIC, ‘best-subset’ iteration) to determine which combination of independent variables best predicted each community descriptor at the subplot, plot, and site scale. Like most regression analyses, AIC modelling can be strongly influenced by collinearity among variables [67]. Our data set is replete with correlated variables, since the percent cover indices among bank vegetation types, among substrate types, and among landform types are strongly correlated. So, rather than using these correlated variables in the AIC models, we conducted principal component analyses (PCA) on the independent variables and conducted the AIC analysis on the loadings for principal components with eigenvalues > 1 [68]. 

For these analyses, only samples with non-zero values for that dependent variable were included in the analyses. So, for example, although 300 subplots were sampled (10 subplots per plot × 2 plots per site × 15 sites = 300), adult odonates occurred at only 221 of these subplots. Consequently, only the environmental conditions at these 221 subplots were used in the PCA analyses for adult odonate abundance, richness, and diversity at the subplot scale. We only included non-zero samples for two reasons. First, we consider a value of zero for richness and diversity as ‘undefined’, rather than a quantitative metric. They are descriptors of an assemblage, but if there are no individuals present, then there is no assemblage to describe. The same is not true for abundance, obviously, but we chose to exclude samples with zero abundance for a different reason. Because we sampled at such a small scale (2 m × 2 m), the absence of individuals may not only indicate habitat avoidance, but just low density. If there are fewer than 10 individuals in a plot, then at least one subplot must be empty, regardless of habitat preferences. So, non-zero values give a more conservative description of the response to environmental variation. At the plot and sites scales, sample sizes for richness analyses may exceed those for abundance and diversity because, for example, a species may be observed outside of an instantaneous subplot sample, in a plot that otherwise had no observations. That plot would have a non-zero value for richness and would be included in that analysis, but the abundance and diversity values (calculated on subplot sampling totals) would be zero—excluding this plot from those analyses.

Although generating and analyzing principal components addresses the problem of collinearity among variables, it can exaggerate relationships between the original independent variables and the dependent variables. For example, an independent variable may be significantly correlated with a principle component axis, the principal component may account for a significant fraction of variation in the matrix of environmental variables, the principal component may be a significant predictor in an AIC model, but, the direct correlation between that independent variable and the dependent variable may be insignificant. So, we interpreted these results in the following manner. First, we identified the principal components that significantly predict a dependent variable in an AIC model. Then, we identified the independent variables that significantly correlated with these predictive principal components (subplot scale: r > |0.400|, *p* < 0.001; plot scale: r > |0.400|, *p* < 0.02; site scale: r > |0.512|, *p* < 0.05). In tables, these variables are shaded. Finally, we focused on the subset of independent variables that actually had a significant correlation (*p* < 0.05) with the dependent variable. The significant correlation coefficients are reported directly in the tables. For variables that were not significantly correlated to the dependent variable (*p* > 0.05), but were significantly correlated with a principal component (shaded), only the direction of that relationship with the principal component is presented (as + or -). In addition, several variables—notably water quality and landscape parameters—strongly correlated with downstream position. We used partial correlations, controlling for downstream position, to describe the relationship between the environmental variables and indices of community structure.

Lastly, we described the nested subset composition of the adult odonate, zygopteran, and anisopteran communities at the 15 sites using the NODF method [69], and “Nestedness for Dummies” [70,71], and interpreted these patterns in light of the environmental and spatial relationships among sites. 

## 3. Results

### 3.1. ANOVA and Means

We sampled 590 larvae representing 13 genera, 560 adult damselflies in 9 species, and 54 adult dragonflies in 20 species within the subplots. Only two zygopteran genera were sampled, so suborders were pooled for larval analyses. The mean abundance, richness, and diversity/subplot of larvae, adult odonates, and adult zygopterans varied significantly among sites in nested linear model analyses (‘Site Effect’, *p* < 0.0001, Table 1). There were also significant differences in taxon richness and/or diversity for larvae, adult odonates, and adult zygopterans among plots within sites (‘Plot (Site) Effect’, *p* < 0.05, Table 1). There were no significant differences in mean abundance, richness, or diversity/subplot of adult anisopterans among sites or plots within sites, but there were considerably fewer samples (N = 41, Table 1) distributed across plots and sites in an unbalanced manner. Means and ranges of these dependent variables (Table 2) are consistent with site values from other temperate communities [1,3,35] and much lower than tropical systems [4,22,70]. 

We used AIC models to determine which environmental variables (represented by principal component axes) best explained the variation in larval and adult odonate communities at subplot, plot, and site scales.

### 3.2. Larvae

Larval indices were most strongly related to environmental variation at the subplot scale, predicted by principal components associated with the aquatic variables (as indicated by shaded regions, Table 3). At this scale, all three indices were significantly correlated with the percent cover of detritus, and larval abundance and genus richness were significantly correlated with dissolved oxygen levels (Table 3). Larval abundance was also negatively correlated with the percent cover of bedrock, macrophytes, and cobble, but the relationship with cobble (unshaded) was not associated with a predictive principal component in AIC models (Table 3). 

Larval abundance was negatively correlated with the percent cover of open water, and the percent cover of sunflecks was negatively associated with a significant predictor for all three community indices at this scale (shaded, Table 3). Although the percent cover of wetlands was positively correlated with larval abundance/subplot, it was unrelated to a predictive variable in the AIC model (unshaded, Table 3). At the plot and site scale, no combination of variables predicted genus diversity. Larval abundance and genus richness remained correlated with the percent cover of detritus at both scales, and the negative relationship with cobble was associated with a significant predictor in AIC models (and significantly correlated with richness) at the site scale (Table 3). Likewise, the percent cover of sunflecks was negatively correlated with genus richness at the plot scale; perhaps related to the associated relationships with the percent cover of forest and developed land (shaded, Table 3).

### 3.3. Adult Odonates

The environmental variables that best explained the variation in odonate community structure changed with spatial scale. Aspects of the bank habitat, such as the percent cover of sun and vegetation (particularly 20–100 cm), were positively associated with all three community indices at the subplot scale and with abundance and richness at the plot scale; but these variables had no effect on mean abundance, richness or diversity of odonates across sites (Table 4). The effects of aquatic variables were also more important at the subplot and plot scales. At the subplot scale, all indices correlated with the percent cover of bedrock and were at least positively associated with the percent cover of aquatic macrophytes and negatively associated with the percent cover of cobble (Table 4). At the plot scale, indices were no longer related to the percent cover of bedrock, but the relationships with macrophytes and cobble remained (Table 4). Physicochemical characteristics and downstream position became more important determinants of odonate richness and diversity as scale increased (Table 4). Further, although the percentage of open water habitat was a significant correlate or predictor to odonate community indices at the subplot and plot levels, no habitat type was significantly correlated with richness or diversity at the site scale—where again, the stronger relationships were with downstream position (Table 4).

When analyzed separately, zygopterans and anisopterans responded differently to these environmental variables across the three spatial scales. At a small scale, zygopteran abundance, richness, and diversity correlated with the percent cover of sunflecks, vegetation, bedrock, pH, and water temperature, and abundance also correlated with the percent cover of development and open water in the landscape (Table 5). At larger spatial scales, there were few predictors of zygopteran abundance. For zygopteran richness and diversity, the relationships with pH and temperature remained significant, the importance of average levels of sunlight, bank vegetation, and landscape types weakened, and downstream position became an important predictor (Table 5).

In contrast, anisopteran abundance, richness, and/or diversity were strongly correlated with the percent cover of sun and bank vegetation (particularly 20–100 cm) at all scales, particularly at the plot scale (Table 6). The percent cover of macrophytes correlated with significant PCA predictors at all scales, and correlated with anisopteran abundance at the site scale (Table 6). With respect to landscape characteristics, all three indices correlated with the percent cover of open water at the subplot and plot scales, and richness was negatively correlated with development and positively correlated with forest and wetland at the site scale (Table 6). Downstream position, which showed increasing importance to zygopteran richness and diversity at larger scales, had no relationship with anisopteran indices at these scales and was negatively correlated with PCA predictors at the subplot scale (Table 6).

Several environmental variables change in a consistent manner downstream (Table 7). For example, the percent cover of detritus and all the physicochemical parameters were all highly correlated with downstream position (Table 7). Likewise, there were significant correlations between downstream position and the percent cover of fields in the landscape, and weak relationships between downstream position and the percent cover of developed and forested land (Table 7). 

Therefore, it is possible that relationships between these variables and community indices are spurious consequences of changes in stream size, depth, flow dynamics, and other intrinsic characteristics that change downstream in this riverine habitat. We conducted partial correlations to describe relationships between environmental variables and community indices while controlling for downstream position (Table 8). So, although there were strong relationships between physicochemical and landscape parameters and odonate (Table 3) and zygopteran (Table 4) indices in AIC analyses, only the relationships with temperature are significant once the confounding effect of downstream position is account for (Table 8). Thus, odonate and damselfly richness and diversity increase downstream, and are only indirectly related to water chemistry and the percent cover different land forms. In contrast, anisopteran abundance and richness remained positively associated with the percent cover of fields and/or wetlands and open water after downstream position is controlled (Table 8). The strong relationship between larval indices and the percent cover of detritus also remained significant in partial correlations, as did the effect of sun and vegetation on certain indices of adult communities (Table 8). 

Odonate richness increased downstream (r = 0.504, *p* < 0.054, N = 15), largely a consequence of adding species to the community (Table 9). This is reflected in a significant nested subset structure (NODF composition subscore = 55.28, Z = 5.915, *p* < 0.001) compared to 500 randomly constructed communities with proportional row and column totals. *Calopteryx maculata* was found at all sites, and the number of *Argia* species increased downstream (Table 9). When analyzed separately, zygopteran richness was strongly correlated with downstream position (Spearman: r = 0.732, *p* = 0.002, N = 15) and communities were more significantly nested (NODF composition subscore = 79.74, Z = 3.922, *p* < 0.001) than the odonate community as a whole. Anisopterans were distributed more sporadically across sites. Riverine species (*Boyeria vinosa, Hagenius brevistylus*, *Progomphus obscurus* and *Macromia* sp.) were joined by various libellulids—habitat generalists that are more common on ponds and lakes. Although anisopteran communities were also significantly nested (NODF composition subscore = 41.93, Z = 2.94, *p* < 0.01), richness was not correlated with downstream position of sites (Spearman: r = −0.176, *p* > 0.05, N = 13). 

## 4. Discussion

The goal of this study was to describe changes in larval and adult odonate communities, from headwaters in forested suburban areas through downstream sites in an urban landscape, and to determine which environmental variables contributed to these changes at different spatial scales. The novel aspect of this study was an attempt to partition natural variation due to downstream changes in this riverine system from anthropogenic changes in the landscape by including downstream position as an environmental variable. Changes in larval communities were best described by relationships with substrate and water characteristics on a scale of meters. Larval abundance, genus richness, and/or genus diversity were associated with shaded subplots rich in detritus, with mud or sand substrates rather than cobble or bedrock, and high dissolved oxygen. The relationships between genus richness and the percent cover of detritus, sand, and cobble remained significant at larger spatial scales, even after the effect of downstream site position was controlled. These patterns support previous research. Detritus is a refuge from predators and a source of odonate prey [72], and detrital dams are the preferred larval habitat of *Boyeria vinosa* and *Calopteryx maculata* [73], which together represented 20.6% of larvae we collected. Sand substrates are preferred by burrowing odonates such as *Progomphus*, *Stylogomphus*, *Cordulegaster*, *Hylogomphus*, *Phanogomphus*, and *Stylurus* [73,74,75] that comprised 75.6% of the larvae we collected. Further, Brito et al. [50] found that the abundance and richness of libellulid larvae was strongly correlated with dissolved oxygen levels. The distribution and abundance of odonate larvae can certainly be affected by large-scale anthropogenic impacts such as changes in the landscape and pollution [76,77], particularly by impacting stream flow, sediment characteristics, and allochthonous inputs of logs, debris, and detritus [33]. In the Reedy River, however, we conclude that the abundance, richness, and diversity of larval odonate communities are primarily affected by variation in sediment and detrital distributions at a smaller scale.

Adult odonate communities responded in complex ways to changes in the environment. At a small scale, odonate abundance, species richness, and species diversity were positively associated with the percent cover of sunlight, bank vegetation, aquatic macrophytes, bedrock, physicochemical parameters, and open water habitats. These patterns are undoubtedly a consequence of preferences for perches in sunflecks [57] (p. 287), at 20–100 cm [78], or on emergent bedrock. At the plot and site scales, the importance of average bank and substrate conditions declined, and downstream position supplanted landscape effects and explained the relationships between richness and diversity and physicochemical parameters. 

As expected, zygopterans and anisopterans contributed in different ways to these patterns. Zygopteran abundance and richness only significantly correlated with the percent cover of sunflecks, bank vegetation, and bedrock at a small scale. Woodland zygopterans prefer to perch in sunflecks [57] (pp. 287), either to thermoregulate [47], to raise body temperature for courtship [79], or possibly to increase their visibility to territorial intruders and mates. Perching in a sunfleck might be particularly important for cryptic species with reflective spots [57] (pp. 465) or those with structural coloration (like many zygopterans [80]). In contrast, all three indices of anisopteran community structure were more strongly associated with the percent cover of sun and vegetation—particularly aquatic macrophytes and bank vegetation between 20 and 100 cm tall—at the plot and site scales. These patterns are consistent with previous research showing that: (1) anisopterans respond to variation at larger spatial scales as a consequence of greater dispersal capacity [22], and (2) many of the anisopterans in this study prefer to perch at heights between 20 and 100 cm [78]. They also support the hypothesis that anisopterans are more likely to be heliotherms with a warmer thermal niche than zygopterans [47].

The suborders also responded differently to changes in the landscape. Although zygopteran richness and diversity were negatively associated with significant landscape predictors at the plot and site scale, there were no significant relationships with landforms at the site scale once the stronger relationship with downstream position was accounted for in partial correlations. The increase in zygopteran richness downstream is consistent with other studies [34,36,81], although those surveys were conducted at much larger scales and also show dramatic changes in community composition. The lack of a landscape effect is rather surprising, however, because many studies in the tropics have shown that specialist forest zygopterans decline significantly when riparian zones in intact forest are replaced by clearings and agriculture [19,20,21,23,46]. The difference may be a consequence of the long history of deforestation and development in the Eastern USA. All of the zygopterans in the Reedy River are habitat generalists to some degree; we may be describing patterns in a depauperate community that has already lost its forest specialists. 

In contrast, anisopteran indices were unrelated to downstream position and were related to the percent cover of particular landforms at every scale. Abundance correlated with the percent cover of open water habitat at every scale, and richness was positively correlated with the percent cover of wetland and forest (and negatively associated with the percent cover of development) at the site scale. Even after controlling for downstream position in partial correlations, anisopteran abundance or richness was significantly correlated with the percent cover of field and the percent cover of wetlands or open water. These responses are typical for anisopterans and are driven by libellulid habitat generalists that thrive in open, disturbed habitats [10,18,19,24].

The species membership patterns of zygopterans and anisopterans exhibited significant nested subset structure, but different mechanisms were probably responsible. Nestedness can be caused by differential colonization patterns, differential extinction patterns in response to stress, or nested niche space [82,83]. Odonate communities in smaller habitats are often nested subsets of communities inhabiting larger areas; large areas may contain more habitats that support all the species in small isolates of different habitat types [84,85], or large habitats may simply attract more species through increased colonization and the ‘target’ effect of the theory of island biogeography [60,86]. In addition, habitat heterogeneity within sites can contribute to nested subset structure as a consequence of ‘nested niche space’ [87], with homogeneous sites supporting a subset of species also found in more heterogenous sites. In odonates, for example, river drainages with sandy substrates support odonate communities nested within those inhabiting drainages with more varied substrate types [88]. Often, these heterogenous sites contain unique habitats that support particular specialized species, so nestedness analyses can be used to identify species and habitats that need protection [89,90]. Differences in environmental stress across habitats can create nested communities, as the subset of tolerant species that can exploit stressful habitats also may occur in more species-rich communities exploiting benign habitats [91]. In odonates, species using wetlands with short hydro-periods that dry quickly are nested within the communities that use wetlands with longer hydro-periods [92]. Nestedness also occurs among ‘ecological species’ of odonates differing in body size and thermoregulatory ability; small thermoconformers occur in small forest streams, and are nested within communities in larger, sunnier streams and rivers that also contain larger species of heliotherms [47]. 

Because zygopterans are less vagile than anisopterans, their presence at a site is more likely to reflect residency—and true habitat preferences and dependencies—than anisopterans that are more likely to be transient migrants to a site [93]. As such, zygopterans should show nestedness patterns related to environmental stress and nested niche space [93], whereas anisopterans might show nested patterns that are more a consequence of differential colonization and the target effect. Our results support these hypotheses. Zygopteran communities were more strongly nested, and species richness strongly correlated to downstream position. We contend that this is probably a consequence of nested niche space and increasing habitat heterogeneity downstream. *Calopteryx maculata*, *Argia tibialis*, and *Argia fumipennis* prefer small shaded streams [94] (pp. 55,162,157); they dominated communities at headwater sites, but were found in shaded spots at downstream sites that supported additional zygopteran species such as *Argia apicalis*, *Argia moesta*, and *Argia sedula* that more common on wider streams and rivers [94] (pp. 150,153,154). The anisopteran community also exhibited significant nestedness among sites, but richness did not correlate with downstream position. We contend that, as vagile heliotherms, their presence at a site was driven more by habitat openness at the landscape scale (as indicated by the percent cover of field, wetland, and open water in the landscape) rather than the small-scale habitat characteristics that change progressively downstream and drove patterns in zygopteran communities.

## 5. Conclusions

Odonate communities respond to both natural and anthropogenic variation in the environment. In this study, by including downstream position as a variable in analyses, we found that larvae and zygopterans responded to natural environmental variation at a scale of meters. Nestedness patterns in zygopteran communities at a large scale were also more consistent with natural changes in downstream characteristics along the river continuum than direct effects of anthropogenic changes to the landscape. Anisopterans were also strongly responsive to natural variation—particularly in streamside vegetation—at a scale of 10s of meters; probably because of greater dispersal ability. In addition, however, anisopterans were affected by anthropogenic changes to the landscape, and species richness and diversity increased in open areas with fields and wetlands of human origin. Anisopteran communities were also nested, but richness did not correlate with position downstream. It is important to appreciate, however, that these communities may already be depauperate subsets of the broader regional fauna, and that nested subset structure in these communities in suburban and urban landscapes may be a product of the non-random survival of species that can tolerate human impact. This may explain why these communities are more nested than those in tropical forests where turnover between sites—and not nestedness—is the dominant pattern [95]. Broader surveys that include pristine habitats are needed to fully appreciate the relative effects of natural and anthropogenic impacts on odonate community structure in the region. 

## Figures and Tables

**Table 1:** Results analyzing the variation in the abundance, richness, and diversity of larval and adult odonates in 10 subplots within 2 plots at each of 15 sites along the Reedy River, Greenville Co., SC, USA. Only occupied subplots (abundance > 0) are included for each taxon. Abundance values were log_10_ transformed prior to analysis. (“ns” = not statistically significant, *p* > 0.05).

Taxon	Dependent Variable		Site Effect			Plot (Site) Effect		
		N	df	Wald X^2^	*p*	df	Wald X^2^	*p*
Larvae:	Abundance	175	14	117.56	0.0001	14	40.01	0.0001
	Genus Richness	175	14	56.90	0.0001	14	25.82	0.027
	Genus Diversity	175	14	39.97	0.0001	14	18.59	ns
Odonata:	Abundance	221	14	59.57	0.0001	15	23.80	ns
	Species Richness	221	14	63.23	0.0001	15	31.96	0.007
	Species Diversity	221	14	55.54	0.0001	15	26.44	0.034
Zygoptera:	Abundance	217	14	52.16	0.0001	15	18.56	ns
	Species Richness	217	14	49.27	0.0001	15	31.58	0.007
	Species Diversity	217	14	47.41	0.0001	15	29.16	0.015
Anisoptera:	Abundance	41	14	1.38	ns	6	1.40	ns
	Species Richness	41	11	5.80	ns	6	2.88	ns
	Species Diversity	41	11	7.02	ns	6	2.93	ns

**Table 2:** Mean ($\bar{X}$) values and ranges for the abundance, richness, and diversity of larval and adult odonates in 10 subplots within 2 plots at each of 15 sites along the Reedy River, Greenville Co., SC, USA. Only occupied subplots (abundance > 0) are included for each taxon.

Taxon	Dependent Variable	Subplot			Plot			Site		
		N	$\bar{X}$	Range	N	$\bar{X}$	Range	N	$\bar{X}$	Range
Larvae:	Abundance	175	3.4	1–21	29	20.3	1–90	15	39.3	2–135
	Genus Richness	175	1.7	1–5	29	4.6	1–9	15	5.8	2–10
	Genus Diversity	175	1.5	1.0–4.5	29	2.9	1.0–5.5	15	3.3	1.3–4.6
Odonata:	Abundance	221	2.6	1–12	30	20.4	3–44	15	40.8	8–78
	Species Richness	221	1.8	1–6	30	4.8	1–9	15	11.0	1–17
	Species Diversity	221	1.6	1.0–6.0	30	2.5	1.0–4.4	15	2.7	1.0–5.0
Zygoptera:	Abundance	217	2.6	1–12	30	18.6	3–41	15	37.3	8–75
	Species Richness	217	1.6	1–6	30	3.7	1–8	15	6.0	1–9
	Species Diversity	217	1.5	1.0–6.0	30	2.2	1.0–4.2	15	2.4	1.0–4.7
Anisoptera:	Abundance	41	1.3	1–4	18	2.9	1–14	12	4.3	1–17
	Species Richness	41	1.1	1–3	19	1.7	1–4	13	5.7	3–9
	Species Diversity	41	1.1	1.0–2.7	18	1.4	1.0–3.0	12	1.8	1.0–3.0

**Table 3:** Significant correlations between environmental variables and the abundance (“Total”), genus richness (“Rich.”), and Simpson’s Diversity of genera (“Div.”) of larval odonates at three scales. Shading shows variables significantly correlated with significantly predictive principal component axes in AIC models, and the direction of the relationship (+/−) if *p* > 0.05 in independent correlations. The accuracy of AIC models (AIC model r^2^), and the total percentage of the variance in environmental variables of the PCA’s that were significant predictors in the AIC model (% var. sig. predictors) are presented. Negative correlations are in bold italics; significance levels for AIC models and correlation coefficients are: a = *p* < 0.05, b = *p* < 0.01, and c = *p* < 0.001.

Variable	Subplot Scale			Plot Scale			Site Scale		
	Total	Rich.	Div.	Total	Rich.	Div.	Total	Rich.	Div.
N	175	175	175	29	29	29	15	15	15
**AIC model r^2^**	0.14 ^c^	0.14 ^c^	0.07 ^b^	0.29 ^b^	0.46 ^c^	0.06	0.48 ^a^	0.24 ^a^	0.0
**% var. sig. predictors**	23.1	22.0	16.3	13.1	21.0	−	25.1	12.8	−
*Bank Variables:*									
% sun	−	−	−	−	***0.401*** ^a^		−		
% no vegetation			+				−	−	
% veg. < 20 cm				+	+				
% veg. 20–100 cm									
% veg. 100–200 cm					+		+	+	
% veg. 200–300 cm				+	+				
*Aquatic Variables:*									
% bedrock	***0.154*** ^a^	−	−		***0.461*** ^a^				
% cobble	***0.160*** ^a^						−	***0.609*** ^a^	
% gravel	−				+		−		
% sand		+					+	+	
% mud		0.158 ^a^							
% macrophytes	***0.173*** ^a^	−	−				+		
% algae	−	−	−						
% detritus	0.196 ^b^	0.250 ^c^	0.208 ^b^	0.549 ^b^	0.592 ^c^		0.763 ^c^	0.685 ^c^	
pH									
Temperature									
Conductivity									
Dissolved oxygen	0.307 ^c^	0.211^b^	+						
*Landscape Variables:*									
Downstream position									
% Developed					+				
% Forested					−				
% Field									
% Wetland	0.218 ^b^						+	+	
% Open water	***0.182*** ^a^	−	−				−		

**Table 4:** Significant correlations between environmental variables and the abundance (“Total”), species richness (“Rich.”), and Simpson’s Diversity (“Div.”) of adult odonates at three scales. Shading shows variables significantly correlated with significantly predictive principal component axes in AIC models, and the direction of the relationship (+/−) if *p* > 0.05 in independent correlations. The accuracy of AIC models (AIC model r^2^), and the total percentage of the variance in environmental variables of the PCA’s that were significant predictors in the AIC model (% var. sig. predictors) are presented. Negative correlations are in bold italics; significance levels for AIC models and correlation coefficients are: a = *p* < 0.05, b = *p* < 0.01, and c = *p* < 0.001.

Adult Odonates	Subplot			Plot			Site		
	Total	Rich.	Div.	Total	Rich.	Div.	Total	Rich.	Div.
N	221	221	221	30	30	30	15	15	15
**AIC model r^2^**	0.142 ^c^	0.170 ^c^	0.155 ^c^	0.310 ^b^	0.740 ^c^	0.728 ^c^	0.00	0.565	0.629
**% var. sig. predictors**	15.8	38.1	45.0	11.7	65.0	46.9	−	27.0	17.1
*Bank Variables:*									
% sun	0.376 ^c^	0.315 ^c^	0.262 ^c^	0.473 ^b^	+	+			
% no vegetation	***0.208*** ^b^	***0.198*** ^b^	***0.155*** ^a^		***0.398*** ^a^	−			
% veg. < 20 cm	0.150 ^a^								
% veg. 20–100 cm	+	+	+		0.418 ^a^	+			
% veg. 100–200 cm					−				
% veg. 200–300 cm						+			
*Aquatic Variables:*									
% bedrock	0.192 ^b^	0.253 ^c^	0.253 ^c^					−	
% cobble					−				
% gravel	−	***0.157*** ^a^	***0.153*** ^a^	***0.404*** ^a^	***0.428*** ^a^	***0.415*** ^a^		***0.565*** ^a^	
% sand			−		−				
% mud			+		0.395 ^a^	0.467 ^b^		0.535 ^a^	
% macrophytes	+	0.165 ^a^	0.171 ^a^	+	0.372 ^a^	+			
% algae									
% detritus		−	−	−	+	+			
pH		0.197 ^b^	0.226 ^c^	0.401 ^a^	0.469 ^b^	0.644 ^c^		+	0.698 ^b^
Temperature	0.223 ^c^	0.293 ^c^	0.294 ^c^	0.554 ^b^	0.736 ^c^	0.693 ^c^		0.804 ^c^	0.782 ^c^
Conductivity		+	+		+	0.524 ^c^		+	0.629 ^a^
Dissolved oxygen					+	+		+	
*Landscape Variables:*									
Downstream position		+	+		0.442 ^a^	0.573 ^c^		0.507 ^a^	0.644 ^b^
% Developed					+				
% Forested	***0.180*** ^b^				−				
% Field		−	−		−	−		−	−
% Wetland					+				
% Open water	0.258 ^c^	0.288 ^c^	0.259 ^c^	0.394 ^a^	0.471 ^b^	+			

**Table 5:** Significant correlations between environmental variables and the abundance (“Total”), species richness (“Rich.”), and Simpson’s Diversity (“Div.”) of adult zygopterans at three scales. Shading shows variables significantly correlated with significantly predictive principal component axes in AIC models, and the direction of the relationship (+/−) if *p* > 0.05 in independent correlations. The accuracy of AIC models (AIC model r^2^), and the total percentage of the variance in environmental variables of the PCA’s that were significant predictors in the AIC model (% var. sig. predictors) are also presented. Negative correlations are in bold italics; significance levels for AIC models and correlation coefficients are: a = *p* < 0.05, b = *p* < 0.01, and c = *p* < 0.001.

Adult Zygoptera	Subplot			Plot			Site		
	Total	Rich.	Div.	Total	Rich.	Div.	Total	Rich.	Div.
N	217	217	217	30	30	30	15	15	15
**AIC model r^2^**	0.108 ^c^	0.082 ^c^	0.077 ^c^	0.248 ^a^	0.482 ^c^	0.481^c^	0.00	0.865 ^c^	0.524 ^c^
**% var. sig. predictors**	29.8	38.0	38.0	11.7	23.5	27.6	−	63.6	17.1
*Bank Variables:*									
% sun	0.281 ^c^	0.165 ^a^	+	+				−	
% no vegetation	***0.166*** ^a^	−	−		−			+	
% veg. < 20 cm									
% veg. 20–100 cm	+	−	−		+			+	
% veg. 100–200 cm									
% veg. 200–300 cm						+		−	
*Aquatic Variables:*									
% bedrock	0.190 ^b^	0.263 ^c^	0.269 ^c^					+	
% cobble								−	
% gravel	−	−	−	***0.373*** ^a^		***0.382*** ^a^		−	
% sand		−	−						
% mud	+	+	+			+		+	
% macrophytes				+				+	
% algae									
% detritus				+				+	
pH		0.236 ^c^	0.261 ^c^	0.418 ^a^	0.546 ^b^	0.675 ^c^		0.631 ^a^	0.745 ^b^
Temperature	0.179 ^b^	0.239 ^c^	0.242 ^c^	0.510 ^b^	0.757 ^c^	0.630 ^c^		0.856 ^c^	0.731 ^b^
Conductivity		−	+		+	+			0.517 ^a^
Dissolved oxygen					+	+		+	
*Landscape Variables:*									
Downstream position		+	+		0.513 ^b^	0.536 ^b^		0.722 ^b^	0.640 ^a^
% Developed	0.136 ^a^							+	
% Forested	***0.191*** ^b^							−	
% Field	−	−	−		−	***0.436*** ^a^		***0.564*** ^a^	−
% Wetland	−							+	
% Open water	0.150 ^a^			+					

**Table 6:** Significant correlations between environmental variables and the abundance (“Total”), species richness (“Rich.”), and Simpson’s Diversity (“Div.”) of adult anisopterans at three scales. Shading shows variables significantly correlated with significantly predictive principal component axes in AIC models, and the direction of the relationship (+/−) if *p* > 0.05 in independent correlations. The accuracy of AIC models (AIC model r^2^), and the total percentage of the variance in environmental variables of the PCA’s that were significant predictors in the AIC model (% var. sig. predictors) are presented. Negative correlations are in bold italics; significance levels for AIC models and correlation coefficients are: a = *p* < 0.05, b = *p* < 0.01, and c = *p* < 0.001.

Adult Anisoptera	Subplot			Plot			Site		
	Total	Rich.	Div.	Total	Rich.	Div.	Total	Rich.	Div.
N	41	41	41	18	19	18	12	13	12
**AIC model r^2^**	0.116 ^a^	0.088 ^a^	0.080 ^a^	0.517 ^b^	0.171 ^a^	0.495 ^b^	0.813 ^b^	0.401 ^a^	0.553 ^a^
**% var. sig. predictors**	21.1	21.1	21.1	26.2	18.0	26.0	46.5	13.4	8.6
*Bank Variables:*									
% sun	0.352 ^a^	+	+	0.849 ^c^	+	+	0.813 ^b^		
% no vegetation	−	−	−	−		***0.687*** ^b^			***0.851*** ^c^
% veg. < 20 cm				−		+	+		
% veg. 20–100 cm	+	+	+	0.504 ^a^	0.521 ^a^	+	0.588 ^a^		
% veg. 100–200 cm				−		+	+		0.595 ^a^
% veg. 200–300 cm				***0.531*** ^a^	−		−		
*Aquatic Variables:*									
% bedrock								−	
% cobble							−		
% gravel						−			
% sand						+	+		
% mud									
% macrophytes	+	+	+	+		+	0.687 ^a^		
% algae	+	+	+	+		+			
% detritus	***0.323*** ^a^	−	−	−		−	+		
pH									
Temperature				+		+			
Conductivity									
Dissolved oxygen									+
*Landscape Variables:*									
Downstream position	−	−	−						
% Developed	−	−	−					***0.711*** ^b^	
% Forested								0.556 ^a^	
% Field	+	+	+	+		+		+	
% Wetland							+	0.674 ^a^	
% Open water	0.392 ^a^	0.330 ^a^	0.339 ^a^	0.698 ^c^	0.617 ^b^	0.653 ^b^	0.704 ^a^		

**Table 7:** Mean (± 1 standard deviation (s.d.)) and range of environmental variables for 15 sites along the Reedy River in Greenville, SC, USA, and the Spearman rank correlation of site means with downstream position (*p* = significance level; ns = *p* > 0.1).

Variable	Mean ± 1 s.d.	Range	Correlation	*p*
*Bank Variables:*				
% sun	30.4 ± 18.0	11.0–82.1	−0.084	ns
% no vegetation	35.3 ± 18.9	4.2–73.1	0.272	ns
% veg. < 20 cm	14.8 ± 9.2	2.0–35.9	0.114	ns
% veg. 20–100 cm	23.5 ± 12.8	7.9–52.8	0.184	ns
% veg. 100–200 cm	19.5 ± 17.9	0.7–62.6	−0.488	0.065
% veg. 200–300 cm	6.8 ± 4.7	0.2–19.9	0.517	0.048
*Aquatic Variables:*				
% bedrock	10.7 ± 14.2	0.00–41.5	0.249	ns
% cobble	8.8 ± 15.8	0.00–49.5	−0.129	ns
% gravel	7.1 ± 11.2	0.00–32.0	0.114	ns
% sand	52.8 ± 17.8	16.079.8	−0.168	ns
% mud	6.13 ± 6.7	0.0–22.9	0.325	ns
% macrophytes	3.1 ± 6.0	0.0–24.0	−0.092	ns
% algae	1.3 ± 2.6	0.0–8.5	0.193	ns
% detritus	10.8 ± 7.5	1.5–29.6	0.522	0.046
pH	6.6 ± 0.3	6.1–7.3	0.893	0.001
Temperature	23.2 ± 2.7	17.1–27.5	0.714	0.003
Conductivity	78.9 ± 37.1	51.0–205.2	0.873	0.001
Dissolved oxygen	4.7 ± 1.2	1.7–6.5	−0.577	0.024
*Landscape Variables:*				
% Developed	63.4 ± 26.1	23.4–99.3	0.501	0.057
% Forested	20.5 ± 14.3	0.2–42.7	−0.470	0.077
% Field	8.1 ± 9.1	0.0–32.9	−0.819	0.001
% Wetland	7.1 ± 9.4	0.0–31.6	−0.151	ns
% Open water	0.2 ± 0.1	0.0–1.7	−0.060	ns

**Table 8:** Summary of significant partial correlations (*p* < 0.05), controlling for downstream site position, between mean environmental variables at 15 sites along the Reedy River, Greenville, SC, USA, and the abundance, richness, and diversity of odonate larvae and adults. (Negative correlations are in bold italics.)

Variable	Larvae			Odonata			Zygoptera			Anisoptera		
	Tot.	R	Div.	Tot.	R	Div.	Tot.	R	Div.	Tot.	R	Div.
**df**	12	12	12	12	12	12	12	12	12	9	10	9
*Bank Variables:*												
% sun										0.872		
% no vegetation				***0.606***	***0.582***		***0.535***	***0.611***				***0.838***
% veg. < 20 cm												
% veg. 20–100 cm										0.607		
% veg. 100–200 cm												
% veg. 200–300 cm												
*Aquatic Variables:*												
% bedrock												
% cobble		***0.619***									***0.675***	
% gravel					***0.658***	***0.626***		***0.721***	***0.552***			
% sand		0.543										
% mud												
% macrophytes										0.720		
% algae												
% detritus	0.865	0.665										
pH												
Temperature				0.647	0.729	0.608	0.583	0.713				
Conductivity												
Dissolved oxygen												
*Landscape Variables:*												
% Developed												
% Forested												
% Field										0.742	0.667	
% Wetland											0.607	
% Open water										0.707		

**Table 9:** The presence-absence matrix of odonate adults seen at 15 sites along the Reedy River in Greenville, SC, USA, ordered by species richness (‘Richness’, columns) and species frequency (‘Fr.’, rows) to maximally ‘pack’ the matrix. Species richness is significantly correlated with downstream position (‘Downstream Pos.’, r = 0.570, N = 15, *p* < 0.05). Sites listed from left to right: CN = Lake Conestee Nature Preserve; HS = Hudson Street; CP = Cleveland Park; SR = Swamp Rabbit Bakery; WB = Watkins Bridge; GV = Green Valley Country Club; AP = Andover Park; RB = Riverbend Equestrian Park; SN = South Pleasantburg Nursery; SS = Sulphur Spring; FP = Falls Park; MP = Mayberry Park; SP = Spring Park; PP = Poinsett Park; VH = Vista House.

Site:	CN	HS	CP	SR	WB	GV	AP	RB	SN	SS	FP	MP	SP	PP	VH	
**Downstream Pos.:**	13	8	10	6	4	2	11	3	12	5	9	7	1	1	2	
**Richness:**	16	14	13	13	13	13	12	12	10	10	9	8	5	2	1	
**Species**																**Fr.**
*Calopteryx maculata*	1	1	1	1	1	1	1	1	1	1	1	1	1	1	1	15
*Argia tibialis*	1	1	1	1	1	0	1	1	1	1	1	1	0	1	0	12
*Argia fumipennis*	1	1	1	1	1	1	1	1	0	1	1	1	1	0	0	12
*Argia moesta*	1	1	1	1	1	1	1	1	1	1	1	1	0	0	0	12
*Hagenius brevistylus*	1	1	1	1	1	1	1	0	1	0	1	1	0	0	0	10
*Argia sedula*	1	1	1	1	0	1	1	0	1	0	1	1	0	0	0	9
*Argia apicalis*	1	1	1	1	0	0	1	1	1	0	1	0	0	0	0	8
*Libellula incesta*	1	1	1	0	1	1	1	1	0	0	1	0	0	0	0	8
*Hetaerina americana*	1	1	1	1	1	0	1	0	1	0	0	1	0	0	0	8
*Boyeria vinosa*	0	1	0	1	1	0	1	1	1	1	0	1	0	0	0	8
*Plathemis lydia*	1	1	0	0	1	0	0	1	1	1	0	0	1	0	0	7
*Erythemis simplicicollis*	1	0	0	1	1	1	0	0	0	1	0	0	1	0	0	6
*Pachydiplax longipennis*	1	0	0	1	1	1	0	1	0	1	0	0	0	0	0	6
*Progomphus obscurus*	0	0	1	0	1	0	1	0	0	0	1	0	0	0	0	4
*Ischnura posita*	1	0	1	1	0	1	0	0	0	0	0	0	0	0	0	4
*Perithemis tenera*	0	1	1	1	0	1	0	0	0	0	0	0	0	0	0	4
*Libellula vibrans*	1	0	1	0	0	0	0	1	0	0	0	0	0	0	0	3
*Hetaerina titia*	0	1	0	0	0	0	0	0	0	1	0	0	0	0	0	2
*Libellula cyanea*	0	0	0	0	0	1	0	1	0	0	0	0	0	0	0	2
*Libellula luctuosa*	1	0	0	0	0	1	0	0	0	0	0	0	0	0	0	2
*Dythemis velox*	0	0	0	0	0	0	0	0	0	0	0	0	1	0	0	1
*Tramea carolina*	0	0	0	0	0	0	0	0	0	1	0	0	0	0	0	1
*Dromogomphus spinosus*	0	0	0	0	0	0	0	0	1	0	0	0	0	0	0	1
*Nannothemis bella*	0	0	0	0	0	0	0	1	0	0	0	0	0	0	0	1
*Libellula axilena*	0	0	0	0	0	0	1	0	0	0	0	0	0	0	0	1
*Macromia sp.*	0	0	0	0	0	1	0	0	0	0	0	0	0	0	0	1
*Somatochlora tenebrosa*	0	0	0	0	1	0	0	0	0	0	0	0	0	0	0	1
*Celithemis eponina*	0	1	0	0	0	0	0	0	0	0	0	0	0	0	0	1
*Orthemis ferruginea*	1	0	0	0	0	0	0	0	0	0	0	0	0	0	0	1
